# Prevalence of pain and its association with quality of life of patients with heart failure in a developing country: findings from a multicenter cross-sectional study

**DOI:** 10.1186/s12872-022-02864-7

**Published:** 2022-09-28

**Authors:** Deema Mhesin, Hadeel Nazzal, Jalilah Amerah, Murad Azamtta, Yahia Ismail, Yunis Daralammouri, Mazen A. Abdalla, Mohammad M. Jaber, Amer A. Koni, Sa’ed H. Zyoud

**Affiliations:** 1 Department of Medicine, College of Medicine and Health Sciences, An-Najah National University, Nablus, 44839 Palestine; 2 Department of Cardiology, An-Najah National University Hospital, Nablus, 44839 Palestine; 3 Department of Orthopedics, An-Najah National University Hospital, Nablus, 44839 Palestine; 4 Department of Clinical and Community Pharmacy, College of Medicine and Health Sciences, An-Najah National University, Nablus, 44839 Palestine; 5 Division of Clinical Pharmacy, Department of Hematology and Oncology, An-Najah National University Hospital, Nablus, 44839 Palestine; 6 Poison Control and Drug Information Center (PCDIC), College of Medicine and Health Sciences, An-Najah National University, Nablus, 44839 Palestine; 7 Clinical Research Center, An-Najah National University Hospital, Nablus, 44839 Palestine

**Keywords:** Pain, Prevalence, Heart failure, HRQoL, Quality of life, 5Q-5D, Palestine

## Abstract

**Background:**

Heart failure (HF) is considered one of the main causes of morbidity and death among chronic diseases worldwide. Patients have increasingly reported chronic pain in long-standing heart failure as a disturbing symptom. Its unknown etiology and mechanism, in addition to its insidious progressive nature, made both the doctor and the patient not notice it until it affects the quality of life (QoL) and general health status. The primary objective of this study is to find the prevalence of pain in chronic heart failure patients and its impact on their QoL. The secondary objective is to determine the predictors of QoL in HF patients.

**Methods:**

A multicenter cross-sectional design was used. The European Quality of Life scale five dimensions scale and the Brief Pain Inventory were adopted to evaluate QoL and pain, respectively. The Statistical Package for the Social Sciences version 25 was applied to present the data. The Mann-Whitney U, Kruskal-Wallis, and Cronbach alpha tests were used.

**Results:**

The final study had a total of 142 individuals. The prevalence of pain among HF patients was 84.5%. Knee pain was the main complaint among patients. Our patients' median pain severity score was 18 [5.00–25.00], while the median pain interference score was 39 [24.75–53.00]. They had a median EQ-5D score of 0.34 [0.0–0.6] and an EQ-VAS score of 50 [30–70]. Pain severity (*p* = 0.004 and *p* < 0.001, respectively) and pain interference (*p* < 0.001 and *p* = 0.001, respectively) were found to significantly associated with both QoL scores; the visual analogue scale (EQ-VAS) and EQ-5D-5L. In multivariate analysis, monthly income was the only variable significantly correlated with EQ-VAS and EQ-5D-5L, along with pain variables.

**Conclusions:**

Pain is a common symptom among patients with HF and is significantly associated with their QoL. Low income is also highly associated with poor QoL. Definitive guidelines should be achieved to increase awareness and understanding of the importance of pain management, reaching a higher QoL level, less pain, and good adherence to HF medications.

## Background

Heart failure (HF) is a group of signs and symptoms caused by a weakened heart, resulting in decreased longevity [1]. Typical symptoms describe it (that is, breathlessness) and signs (that is, peripheral edema) caused by a functional and/or structural heart abnormality [2]. When these symptoms progress, affecting the patient’s long-term quality of life (QoL), then it is called chronic heart failure (CHF) [3]. HF is a common disease, with approximately 37.7 million people living worldwide [4]. Consequently, HF is one of the most common causes of morbidity and death in chronic diseases worldwide [5].

According to the International Association for the Study of Pain (IASP), the updated and recent definition of pain is an unpleasant sensory and emotional experience associated with, or resembling that associated with, actual or potential tissue damage [6].

Quality of life is a term that aims to describe a population's or individual's entire well-being throughout their life at a specific point in time, incorporating both positive and negative aspects. For example, personal health (physical, mental, and spiritual), relationships, education level, work environment, social standing, money, and a sense of security are all important parts of QoL [7]. However, Health-related quality of life (HRQoL) is how well an individual functions in their life and their perceived well-being in mental, physical, and social aspects of health [8].

HF disease progression is characterized mainly by the decline in daily patient activities and its effect on the QoL [9]. However, it turned out that even in the asymptomatic periods of the disease [5, 10], these changes include a wide variety of deteriorations and limitations in performing basic activities, in addition to daytime and nighttime sleep [11], anxiety, and depression. This impact is not directly related to fatigue and dyspnea, which are the main symptoms of HF, but rather to the unrecognized pain by patients and clinicians [10, 12, 13]. Furthermore, it is rare for patients with HF to present with only one symptom. Instead, they mostly present with multiple symptoms together, and pain is one of them [13, 14]. This under-recognition of pain was attributed to the fact that this pain is insidious and, therefore, could not be noticed immediately [5].

Chronic pain appears to be prevalent, between 23 and 85% among patients [5]. However, this pain is not uniformly described by them. Its nature is not well understood and varies in intensity from patient to patient, but some patients describe their pain as localized or generalized [12]. From another perspective, chronic HF pain is suggested to be of different origins but can be classified into classes according to the mechanism, such as neuropathic, inflammatory, and ischemic pain [5].

Regardless of the nature of pain, the etiology is still vague and controversial. Several factors play a major role in pain severity, including age, comorbidities, mental health status, and even a history of cancer [5]. However, an essential factor found to have the greatest role in the severity of pain is the ejection fraction. The lower the ejection fraction, the more severe the pain [12, 15].

Heart failure symptoms were approved to be associated QoL [16, 17]. Nevertheless, pain is influenced by several factors, and cause and effect is a complex matter. In addition, it is quite difficult to relate a symptom directly to heart failure, as these patients have symptom clusters and the correlation depends on the symptom characteristics studied and the prevalence of these symptoms [18].

As a result of a previous review article, the prevalence of pain in HF patients ranges between 23 and 85% [5]. A wide-range prevalence like this cannot be neglected to improve treatment, and this improvement starts by considering pain as a significant symptom of HF. In addition, some studies have discovered that several symptoms are associated with lower QoL in HF patients. Pain, anxiety, and depression, for example, were all associated with a lower QoL [19]. Although recent evidence suggests that a high proportion of people with HF experience pain and correlate with a lower QoL [20], we do not know how pain affects QoL.

Furthermore, there is strong evidence that there is a correlation between hidden and unrecognized pain and decreased QoL in HF patients [13]. This study provides good insight into the factors related to QoL. Therefore, this study aims to look for the pain to determine its prevalence and severity in HF patients from a developing country and better understand its association with QoL. It also aims to show the contributions between these areas to establish medical management composed of a multidisciplinary team to control this essential symptom.

## Methods

### Study design

A multicenter cross-sectional design was used, including samples from the cardiology department at Al-Watani Hospital, An-Najah National University Hospital, and the Ministry of Health Clinics in Nablus, Palestine, from November 2020 to March 2021. Due to the coronavirus disease 2019 (COVID-19) crisis, many patients stopped visiting hospitals and clinics as they used to do before, so we visited them at their houses to complete the sample.

### Sample size and sampling procedure

6th-year medical students carried out data collection via face-to-face interviews. A convenience sample of 142 patients met the criteria and took part in the analysis. Frequent check-ups have been performed to reassure the sampling environment, clear definitions, and criteria correspondence.

### Inclusion criteria

We included patients 18 years old or more, who had chronic HF for more than 3 months, were stable on the treatment regimen, having a left ventricular ejection fraction of < 40% determined by echocardiography or ventriculography, with the mental and physical capacity to speak with the interviewer and the ability to collect all clinical and demographic data. Those who refused to give their consent, those who were unstable. And patients with documented lacking cognitive ability to respond to the data collection procedures were also excluded. We also excluded patients who had missing items on the BPI scale.

### Data collection instrument

Data were obtained through multiple questionnaires. The main variables used were: (i) Sociodemographic data: including age, gender, body mass index (BMI), residency, education status, monthly income, social status, and job status (ii) Clinical data related to HF, including duration of HF disease, comorbid diseases, and chronic medications obtained from the medical record, (iii) HRQoL of HF patients, and (iv) Pain-related data. The data collection form was built based on previous studies' information [5, 9, 10, 12].

### HRQoL measurement

The 5-dimensional European Quality of Life scale (EQ5D) is used to assess the HRQoL of the patient. It is a general instrument that allows the comparison of the HRQoL evaluation findings between different populations. The EQ5D instrument includes a descriptive system and a visual analogue one. The EQ5D5L system is the descriptive one. Mobility, pain/discomfort, self-care, anxiety/depression, and typical activities are the dimensions of this system. Each component has five levels: no issues, minor issues, moderate issues, severe issues, and extreme issues. The patient should provide the most appropriate answer in each of the five components to reflect his or her health status. This option yields a one-digit number indicating the level chosen for that dimension. Then the five-dimensional numbers can be merged to form a five-digit number that indicates the patient's health status. The visual analogue scale (EQ-VAS) is a thermometer-like scale that asks patients to rate their health status on the day of questionnaire completion. Zero denotes the worst health and 100 represents the best health [20]. Cohen’s k values for EQ-5D mobility, self-care, usual activities, pain/discomfort, and anxiety/depression items were 0.66, 1.0, 0.48, 0.66, and 0.48, respectively (*p* ≤ 0.001 for all dimensions). Moreover, Cronbach’s alpha was 0.75 for the Arabic version of the EQ-5D. The intra-class correlation coefficient for the EQ-VAS was 0.78 [21]. The Euro-QoL Research Foundation allowed us to utilize the Arabic form of the EQ-5D (registered ID: 41,390).

### Measurement of pain

We define the prevalence of pain by the yes/no question [22]. The Brief Pain Inventory (BPI) in the Arabic version is the tool that was applied to evaluate the patient's severity of pain and the extent to which pain interferes with his ability to function in daily life. The BPI has two categories: Pain Interference and Pain Intensity. The two components of pain interference help uncover several issues that must be addressed to treat the patient accurately. Activity interference and affective interference are the two dimensions in question. The term "activity interference" refers to any type of activity, including more physical activities like walking. Affective interference is concerned with internal or emotional components of daily living (for example, happiness). Pain interference is divided into seven: mood, walking, working, general activity, sleeping, relationships, and enjoying life [23]. Each of them received a score ranging from 0 to 10, and a pain interference score was calculated by summing up the marks earned on the seven questions, the resultant score was between 0 and 70 [22]. Patients with a total 10-point score of ≤ 5 were considered ‘low interference’, while those who have a score of > 5 were marked as ‘high interference’ [24].

However, the following issues are evaluated in the items on pain severity: The worst pain experienced in the previous 24 h, the least pain experienced in the previous 24 h, the average pain experienced in the previous 24 h, and the pain experienced at the time of evaluation. Participants received a score ranging from zero to ten for each of the statements mentioned above. After summing up, the resultant pain severity scores ranged from 0 and 40 [22]. Specifically, the total score ranges between 0 and 40, which was converted into a 10-point scale. A score of ≤ 4 was considered mild, > 4–6 was moderate, and > 6 was severe [24]. In addition, a picture of the human body is shown, which allows the patient to point to pain sites that he complains of. We presented the sites of pain according to the BPI scale. The scale also asks about the patient's pain management and effectiveness. Cronbach alpha for the interference items 0.92 and 0.82 for the severity. Correlations between the severity and interference items ranged between 0.25 and 0.57 (*P* < 0.05) [25]. We were granted permission to use the MD Anderson Cancer Center's Arabic BPI-Short Form version, which had already been translated and verified [25, 26].

Before the interviewer, we explained to patients that all questions were related to heart failure, and they had to answer accordingly.

### Confidentiality

Written informed consent was obtained from all patients and informed that all data are confidential and will be used only for clinical research and publication.

### Statistical analysis

We used version 25 of the Statistical Package for the Social Sciences (SPSS) (IBM SPSS Statistics for Windows, IBM Corporation, Armonk, NY) for analysis. Sociodemographic and clinical factors were described using descriptive analysis. The normality of the data was checked using the Kolmogorov-Smirnov test. Their frequencies and percentages represented categorical variables. Continuous variables were described by means and standard deviations and/or medians and interquartile range. The connections between these characteristics and the QoL scores were then checked using the Mann-Whitney U and Kruskal-Wallis tests. The internal consistency was checked using the Cronbach alpha test. Eventually, regression analysis was used to separate the variables that exhibited a significant link to QoL in bivariate testing. The EQ-5D-5L crosswalk index value calculator (http://www.euroqol.org/about-eq-5d/valuation-of-eq-5d/eq-5d-5l-value-sets.html, accessed 7 May 2021) was used to calculate the EQ-5D-5L score by using values from patients with chronic heart failure. A statistically significant *p*-value was established at < 0.05.

## Results

### Characteristics of the sample

The participants had a mean age of 64.50 ± 10, and 47.2% of them were under the age of 65. Male patients account for around 57% of the overall number of patients, 78.2% are married, and 59.2% live in the city. Most of the patients (82.4%) are unemployed. Additionally, 79.6% of participants earn fewer than 2000 new Israeli Shekels monthly. Most patients receive primary and middle school education with 24.6% and 28.9%, respectively. It should be mentioned that 57% of patients are obese according to their BMI (Table 1).

**Table 1:** Characteristics of the study sample

Variable	Frequency (%) N = 142
*Age (years)*	
< 65	67 (47.2)
65–74	53 (37.3)
≥ 75	22 (15.5)
*Gender*	
Male	81 (57)
Female	61 (34)
*BMI category*	
Healthy weight	17 (12)
Overweight	44 (31)
Obese	81 (57)
*Residency*	
Village	54 (38)
City	84 (59.2)
Camp	4 (2.8)
*Educational level*	
No formal education	18 (12.7)
Primary education	35 (24.6)
Middle school	41 (28.9)
Secondary education	22 (15.5)
University	26 (18.3)
*Marital status*	
Married	111 (78.2)
Single, divorced, widowed	31 (21.8)
*Occupation*	
Employee	25 (17.6)
Unemployed	117 (82.4)
*Monthly income*	
< 2000 NIS	113 (79.6)
≥ 2000 NIS	29 (20.4)
Duration of the disease	
< 4	57 (40.1)
≥ 4	85 (59.9)
*Number of diseases*	
None	3 (2.1)
1	16 (11.3)
2	44 (31)
≥ 3	79 (55.6)
*Chronic medications*	
< 4	13 (9.2)
≥ 4	129 (90.8)

*BMI* Body mass index, *NIS* New Israeli Shekel

### Presence and site of pain

One hundred and forty-two patients with CHF were included in the analysis. 120 (84.5%) of these participants reported chronic pain. Table 2 contains detailed numbers showing the pain sites and presence. Knee pain was the main complaint among patients, with the right predominating (n = 62, 43.7%).

**Table 2:** Presence and site of pain

Variable	Frequency (%) N = 142
*Presence of pain*	
Yes	120 (84.5)
No	22 (15.5)
*Area of pain*	
Head anteriorly	30 (21.1)
Neck anteriorly	12 (8.5)
Right chest anteriorly	27 (19)
Left chest anteriorly	35 (24.6)
Right arm anteriorly	23 (16.2)
Left arm anteriorly	24 (16.9)
Right forearm anteriorly	16 (11.3)
Left forearm anteriorly	17 (12)
Right palm	16 (11.3)
Left palm	18 (12.7)
Right sided abdomen	6 (4.2)
Left sided abdomen	5 (3.5)
Groin	6 (4.2)
Right thigh anteriorly	20 (14.1)
Left thigh anteriorly	18 (12.7)
Right knee	62 (43.7)
Left knee	55 (38.7)
Right leg	21 (14.8)
Left leg	20 (14.1)
Right foot	23 (16.2)
Left foot	23 (16.2)
Head posteriorly	28 (19.7)
Neck posteriorly	6 (4.2)
Right shoulder	27 (19)
Left shoulder	24 (16.9)
Left arm posteriorly	21 (14.8)
Right arm posteriorly	20 (14.1)
Left sided back	40 (28.2)
Right sided back	39 (27.5)
Buttocks and sacral region	6 (4.2)
Right forearm posteriorly	14 (9.9)
Left forearm posteriorly	13 (9.2)
Right dorsum of hand	15 (10.6)
Left dorsum of hand	14 (9.9)
Right thigh posteriorly	16 (11.3)
Left thigh posteriorly	16 (11.3)
Right knee posteriorly	28 (19.7)
Left knee posteriorly	26 (18.3)
Left calf	22 (15.5)
Right calf	22 (15.5)
Left heel	15 (10.6)
Right heel	17 (12.0)
Epigastric	13 (9.2)
Substernal	6 (4.2)
Left ankle	25 (17.6)
Right ankle	24 (16.9)
Left elbow	17 (12.0)
Right elbow	16 (11.3)
Left wrist	27 (19.0)
Right wrist	27 (19.0)
Left hip	9 (6.3)
Right hip	8 (5.6)

### Management of pain

Eighty patients (66.7%) tried medications to relieve pain for patients who had pain, while others relied only on nonpharmacological methods, such as relaxation. About 50% of the participants with pain used acetaminophen. The levels of relief of the subjects differed, with 5% reporting no relief and 12.5% reporting complete relief. Table 3 summarizes the findings on pain management methods.

**Table 3:** Medications used to relieve the pain

Variable	Frequency (%) N = 120
Using of medications	80 (66.7)
Acetaminophen	60 (50)
Metamizole	1 (0.8)
Ibuprofen	10 (8.3)
Diclofenac	7 (5.8)
Tramadol	1 (0.8)
Chlorzoxazone\Paracetamol	1 (0.8)
Meloxicam	1 (0.8)
Dexamethasone	3 (2.5)
Gabapentin	1 (0.8)
Unknown	8 (6.7)
Degree of relief	N = 80
0%	4 (5)
20%	8 (10)
30%	1 (1.3)
40%	4 (5)
50%	8 (10)
60%	14 (17.5)
70%	13 (16.3)
80%	10 (12.5)
90%	8 (10)
100%	10 (12.5)

### Brief invitatory pain

The mean ± SD pain severity score of our patients was 16.5 ± 11.28, while our patients' mean pain interference score was 38.0 with a standard deviation of 18.37. Their medians [Q1–Q3] for both scores were 18 [5.00–25.00] and 39 [24.75–53.00], respectively. The Cronbach alpha test was utilized to calculate the reliability of pain severity and pain interference scores, and the results were 0.886 and 0.836, respectively.

### EQ-VAS and EQ-5D-5L scores

The median EQ-5D score in this cohort of patients was 0.34, with an interquartile range of [0.00–0.65], while the mean ± SD was 0.3 ± 0.36. The reliability of the items tested was 0.75. However, the median EQ-VAS score was 50 [30–70], while the mean ± SD score was 50.8 ± 21.33.

### Univariate and multivariate analysis

Education level (*p* = 0.033) and monthly income (*p* = 0.001) showed a statistically significant association with EQ-VAS scores. Furthermore, it showed a significant association between the EQ-VAS scores and the severity category (*p* = 0.026), with the lowest median 30 (20–52.5) for the severe pain group and with the interference category (*p* < 0.001), the median [Q1–Q3] for low pain interference was 60 (43.75–70) compared to 30 (20–50) for high pain interference.

The other factors were not substantially related to this score (Table 4).

**Table 4:** Visual analogue scores for quality of life-based on socio-demographic and clinical factors

Characteristic	Frequency (%) N = 142	QoL score median (Q1_Q2)	*P *value *
*Age category (years)*			
< 65	67 (47.2)	60 (50–65)	
65–74	53 (37.3)	50 (30–70)	0.402
≥ 75	22 (15.5)	50 (30–70)	
*Educational level*			
No formal education	18 (12.7)	50 (20–70)	
Primary school	35 (24.6)	50 (30–60)	
Middle school	41 (28.9)	50 (30–60)	**0.033**
Secondary school	22 (15.5)	62 (50–70)	
University	26 (18.3)	60 (50–66.3)	
*Gender*			
Male	81 (57)	50 (40–70)	0.719
Female	61 (34	50 (30–67.5)	
*BMI category*			
Healthy weight	17 (12)	60 (30–70)	
Overweight	44 (31)	60 (32.5–70)	0.523
Obese	81 (57)	50 (40–60)	
*Income*			
< 2000	113 (79.6)	50 (30–62.5)	**0.001**
≥ 2000	29 (20.4)	60 (50–75)	
*Residency*			
Village	54 (38)	60 (40–70)	
City	84 (59.2)	50 (32.5–65)	0.728
Camp	4 (2.8)	55 (22.5–91.3)	
*Number of drugs*			
< 4	13 (9.2)	40 (20–72.5)	0.727
≥ 4	129 (90.8)	50 (40–70)	
*Social status*			
Married	111 (78.2)	50 (40–65)	0.692
Single,divorced or widowed	31 (21.8)	60 (30–70)	
*Occupation*			
Employee	25 (17.6)	60 (50–67.5)	0.293
Unemployed	117 (82.4)	50 (30–70)	
*Number of diseases*			
None	3 (2.1)	70 (50–00.0)	
1	16 (11.3)	55 (30–68.75)	0.680
2	44 (31)	52.50 (40–70)	
≥ 3	79 (55.6)	50 (30–65)	
*Duration of the disease*			
< 4	57 (40.1)	40 (20–72.5)	0.272
≥ 4	85 (59.9)	50 (40–70)	
*Pain severity*			
No pain	22 (15.5)	70 (60–76.3)	
Mild pain	59 (41.5)	60 (30–65)	**0.026**
Moderate pain	35 (24.6)	50 (40–60)	
Severe pain	26 (18.3)	30 (20–52.5)	
*Pain interference*			
Low	114 (80.3)	60 (43.75–70)	**< 0.001**
High	28 (19.7)	30 (20–50)	

* Bold values denote statistical significance at the *p* < 0.05 level

The results of the relationships with the EQ-5D-5L are shown in Table 5. There was a statistically significant relationship between education level (*p* = 0.007), gender (*p* = 0.005), social status (*p* = 0.028), occupation status (*p* = 0.002), and income (*p* < 0.001). It also shows a substantial link between EQ-5D-5L and the pain interference (*p* < 0.001) and pain severity (*p* = 0.001) categories. Medians [Q1–Q3] of EQ-5D-5L score for pain severity groups were as follows: 0.74 [0.62–0.84] for no pain, 0.36 [0.02–0.55] for mild pain, 0.18 [− 0.05–0.46] for moderate pain, and 0.04 [− 0.17–0.19] for severe pain. In addition, the median was lower for high pain interference 0.04 [− 0.15–0.15] than low pain interference 0.39 [0.04–0.70].

**Table 5:** Scores for the EQ-5D-5L based on socio-demographic and clinical factors

Variable	Frequency (%) N = 142	EQ-5D-5L score median [Q1_Q2]	*P* value *
*Age category (years)*			
< 65	67 (47.2)	0.43 [− 0.23–0.66]	
65–74	53 (37.3)	0.32 [0.01–0.63]	0.326
≥ 75	22 (15.5)	0.09 [− 0.06–0.50]	
*Educational level*			
No formal education	18 (12.7)	0.07 [− 0.15–0.45]	
Primary school	35 (24.6)	0.18 [0.01–0.47]	**0.007**
Middle school	41 (28.9)	0.29 [0.01–0.66]	
Secondary school	22 (15.5)	0.62 [0.03–0.76]	
University	26 (18.3)	0.40 [0.04–0.70]	
*Gender*			
Male	81 (57.0)	0.36 [0.03–0.72]	**0.005**
Female	61 (34.0)	0.18 [0.05–0.49]	
*BMI category*			
Healthy weight	17 (12.0)	0.22 [− 0.08–0.68]	
Overweight	44 (31.0)	0.46 [0.03–0.71]	0.056
Obese	81 (57)	0.23 [− 0.05–0.57]	
*Income*			
< 2000	113 (79.6)	0.19 [− 0.57–0.60]	** < 0.001**
≥ 2000	29 (20.4)	0.63 [0.35–0.80]	
*Residency*			
Village	54 (38.0)	0.36 [0.04–0.64]	
City	84 (59.2)	0.21 [− 0.41–0.64]	0.535
Camp	4 (2.8)	0.50 [0.12–0.70]	
*Number of drugs*			
< 4	13 (9.2)	0.34 [− 0.06–0.62]	0.584
≥ 4	129 (90.8)	0.34 [0.01–0.65]	
*Social status*			
Married	111 (78.2)	0.36 [0.02–0.68]	**0.028**
Single, divorced or widowed	31 (21.8)	0.11 [− 0.92–0.38]	
*Occupation*			
Employee	25 (17.6)	0.65 [0.35–0.75]	**0.002**
Unemployed	117 (82.4)	0.23 [− 0.15–0.62]	
*Number of diseases*			
None	3 (2.1)	0.55 [0.54–0.00]	
1	16 (11.3)	0.35 [0.01–0.70]	0.334
2	44 (31.0)	0.47 [0.03–0.68]	
≥ 3	79 (55.6)	0.19 [− 0.05–0.55]	
*Duration of the disease*			
< 4	57 (40.1)	0.34 [− 0.06–0.62]	0.584
≥ 4	85 (59.9)	0.34 [0.001–0.65]	
*Pain severity*			
No pain	22 (15.5)	0.74 [0.62–0.84]	
Mild pain	59 (41.5)	0.36 [0.02–0.55]	**0.001**
Moderate pain	35 (24.6)	0.18 [− 0.05–0.46]	
Severe pain	26 (18.3)	0.04 [− 0.17–0.19]	
*Pain interference*			
Low pain	114 (80.3)	0.39 [0.04–0.70]	**< 0.001**
High pain	28 (19.7)	0.04 [− 0.15–0.15]	

* Bold values denote statistical significance at the *p* < 0.05 level

The other variables were not significantly related to this score.

The findings of the linear regression analysis revealed significant positive differences (*p* = 0.02) in EQ-VAS scores related to monthly income. These findings are detailed in Table 6. Both the pain interference score (*p* < 0.001) and the pain severity score (*p* = 0.004) had significant negative associations with the EQ-VAS score.

**Table 6:** Analysis of the relationship between participant characteristics and their quality of life by using multiple linear regression (EQ-vas Score)

Model ^a^		Unstandardized Coefficients		Standardized Coefficients	T	*P* value *	95.0% Confidence Interval for B		Collinearity
		B	Std. Error	Beta			Lower Bound	Upper Bound	VIF
1	(Constant)	68.490	7.630		8.977	< 0.001	53.403	83.577	
	Income (categories)	9.272	3.934	0.176	2.357	**0.020**	1.494	17.051	1.052
	Pain severity (categories)	− 4.971	1.678	− 0.225	− 2.963	**0.004**	− 8.288	− 1.653	1.088
	Pain interference (categories)	− 17.901	4.036	− 0.336	− 4.435	**< 0.001**	− 25.883	− 9.920	1.079

^a^Dependent Variable: VAS QoL

* Bold values denote statistical significance at the *p* < 0.05 level

According to regression analysis, having a lower income level was substantially associated with having a lower EQ-5D score (*p* = 0.044). We discovered a significant negative relationship between the EQ-5D score and both pain interference (*p* = 0.001) and pain intensity (*p* < 0.001) (Table 7).

**Table 7:** Analysis of the relationship between participant characteristics and their quality of life by using multiple linear regression (EQ-5D-Score)

Model ^a^		Unstandardized Coefficients		Standardized Coefficients	T	*P* value *	95.0% Confidence Interval for B		Collinearity
		B	Std. Error	Beta			Lower Bound	Upper Bound	VIF
1	(Constant)	0.760	0.239		3.185	0.002	0.288	1.232	
	Educational level	0.014	0.022	0.049	0.608	0.544	− 0.031	0.058	1.436
	Gender	0.055	0.062	0.075	0.886	0.377	− 0.068	0.177	1.635
	Income	0.142	0.070	0.159	2.037	**0.044**	0.004	0.280	1.375
	Social Status	− 0.049	0.071	− 0.056	− 0.693	0.489	− 0.189	0.091	1.493
	Occupation	− 0.096	0.074	− 0.101	− 1.294	0.198	− 0.242	0.051	1.383
	Pain severity categories	− 0.159	0.029	− 0.424	− 5.575	**< 0.001**	− 0.215	− 0.103	1.308
	Pain interference categories	− 0.226	0.065	− 0.250	− 3.504	**0.001**	− 0.354	− 0.099	1.150

^a^Dependent Variable: EQ-5D-5L index value

* Bold values denote statistical significance at the *p* < 0.05 level

## Discussion

The current work found a high prevalence (84.5%) of pain among Palestinian HF patients. The important findings were significant and negative correlations between pain interference and QoL on the one hand and between pain severity and QoL on the other. Patients with CHF suffer from many symptoms, such as pain. There is a significant relationship between hidden pain and decreased quality of QoL in CHF patients [13]. Several studies have found a variety of symptoms, such as depression and anxiety, that decrease QoL in patients with HF [16, 19, 27–29]. In addition, a large number of publications conducted locally and globally have found a significant correlation between pain and poor QoL in HF patients and other populations [22, 28, 30–36]. As a result, greater attention to this topic is warranted. Importantly, a previous study with a large number of patients with HF from 40 countries revealed that HRQoL was a significant determinant of hospital admissions and all-cause mortality [37]. In addition to emerging evidence, it suggests that a large percentage of people with HF experience pain [28]. Therefore, we adopted the current research to determine the prevalence and sites of pain among patients with HF, their QoL, and the effect of pain and other variables on QoL.

Similar to a previous study, the prevalence of pain among HF patients was 85%, but the percentage of patients who had severe/very severe pain was 42.5%, which was higher than what we found (18.3%) [28]. However, another study on the same topic reported that 48% of HF patients had pain and that pain was not associated with QoL [27]. Differences in findings are probably due to a different instrument being used. Another work revealed a prevalence of 57%, with symptoms like pain associated with lowered performance status [38].

It was reported that 39.5% of HF patients complained of pain from more than one site [39]. Patients complaining of moderate to severe pain have higher adverse cardiac events [40]. When looking at stages, it can be found that 57% had pain in class III compared to 32% in class II. Therefore, pain is a complex issue and its severity and consequences on the patient’s health and life might be overlapped, as there are many factors associated with pain and its severity, such as physical harms, psychological problems, elderly, health literacy, community support, comorbid diseases, religions, and spiritual beliefs [5]. Further research should be conducted to figure out an appropriate way to control pain in chronic HF patients [5].

The current work conducted a detailed assessment of QoL in CHF in Palestine. The BPI scale was used to assess chronic pain symptoms, the EQ-5D scale and its EQ-VAS component were used to evaluate QoL. In our analysis, the median scores for EQ-5D and EQ-VAS were 0.34 and 50, respectively. HF patients were documented to have lower QoL compared with other patients with comorbid conditions and the general public [41]. A large analysis of multiple studies found moderate to high QoL in HF patients in relation to the mental aspect and moderate to poor QoL in the physical aspect [42]. A variety of demographic and clinical variables can have an impact on QoL. Certain demographic variables were associated with a worse QoL during this study, like the female sex. This result is similar to a previous study of 2709 HF patients [43]. But it was different in a study with a smaller sample size [28]. The patient’s age and disease duration were not associated with QoL. However, recent findings revered associations between these factors and QoL [44, 45].

Unemployment and low income were other variables significantly associated with poor QoL. Perhaps this result is because chronic illnesses have many complications, limitations, and acute problems that need a good socioeconomic status to control them [46]. Furthermore, most elderly might have been retired and their access to healthcare could be impaired specifically in a developing country, in addition to handling HF symptoms, which may affect the identifying and management of pain. Another study showed that higher-income people had better knowledge of their condition, healthier habits, preventive measures, and easy access to developed medical centers [47]. Furthermore, we found that single, divorced, or widowed patients had lower QoL than married ones. A previous study confirmed that marriage is associated with higher QoL in anxiety, self-care, cognition, social, sexual domains, and life satisfaction [48]. Furthermore, married patients are less at risk of depression than single or widowed patients [49]. Furthermore, according to our findings, patients with a lower level of education had a poorer QoL than those with a greater level of education. This could be related to differences in understanding the nature of the disease and how it affects QoL [50, 51]. Other publications have revealed that lack of education could contribute to people believing erroneous things about pain, leading them to apply maladaptive coping practices and improper access to pain relief options [52, 53].

The current study will help spread awareness about the importance of pain management in this population in developing countries. Additionally, understanding and managing the effect of chronic pain on the patient's mental health increases the patient's adherence to prescribed HF medications.

## Strengths and limitations

This study has advantages, including the fact that it applied validated questionnaires to examine the prevalence of pain among HF patients and its influence on QoL in a developing country. Furthermore, face-to-face interviews were used to collect data, which may have enhanced the data's reliability. However, there were certain limitations, such as the spread of COVID-19 and the imposition of quarantine, which reduced the sample size and the doctors' strike in government facilities. Furthermore, elderly patients and patients who cannot walk and reach health facilities to receive their medicines and send a family member to replace them made it difficult to contact them. In addition, the most important limitation lies in the fact that its cross-sectional design makes it impossible to build causal links between exposure and outcome variables. Furthermore, the convenience sampling technique may have lowered the study's generalizability to additional HF patients. Importantly, certain clinical and objective variables, such as ejection fraction and treatment of HF were not collected or analyzed, in addition to lacking of detailed information on comorbidities, which certainly have an impact on the occurrence of pain complaints.

## Conclusions

Chronic pain was prevalent among a wide range of patients with stable CHF. The subgroups with the lowest QoL are female gender, low education, low income, and unemployed. Additionally, we found that patients with more pain severity and pain interference had significantly lower QoL than others. Our results will provide policymakers and clinicians with reliable information about the impact of chronic pain on QoL, consider changing policies and taking new steps toward pain management, restrict it in current patients, and prevent its progression in future patients. Definitive guidelines should be established to increase awareness and understanding of the importance of pain management, with special guidelines organizing follow-up visits among those patients to reach a higher QoL level, less pain, and good adherence to HF medication.

## Data Availability

All data from the current work are obtainable from the corresponding author upon request (saedzyoud@yahoo.com).

## Abbreviations

HF: Heart failure
QoL: Quality of life
EQ5D: The European Quality of Life scale 5 dimensions
BPI: The brief pain inventory
SPSS: The statistical package for the social sciences
VAS: The visual analogue scale
CHF: Chronic heart failure
IASP: The International Association for the study of pain
NYHA: The New York Heart Association
COVID-19: Coronavirus disease 2019
BMI: Body mass index
HRQoL: Health-related quality of life
IRB: Institutional review board
